# Pyopneumothorax Caused by Aspergillus terreus and Haemophilus influenzae: A Case Report

**DOI:** 10.7759/cureus.76683

**Published:** 2024-12-31

**Authors:** Joseph Odeyemi, Jessica Emoto, Abdel-Ghanie Abu-Samra

**Affiliations:** 1 Internal Medicine/Pediatrics, University of Kansas School of Medicine, Wichita, USA; 2 Pulmonology/Critical Care, University of Kansas School of Medicine, Wichita, USA

**Keywords:** aspergillus terreus, case report, haemophilus influenzae, hydropneumothorax, pleuropulmonary disease, pyopneumothorax

## Abstract

Empyema, a type of pleural effusion characterized by pus accumulation in the pleural space, is most often caused by bacterial infections, typically as a complication of pneumonia. This case report presents a 70-year-old man with chronic obstructive pulmonary disease (COPD), rheumatoid arthritis, and chronic bilateral hydropneumothoraces, who developed pyopneumothorax due to dual infections with *Haemophilus influenzae* and *Aspergillus terreus*. The patient presented with worsening dyspnea, hypoxemia, and respiratory acidosis, requiring hospitalization and chest tube thoracostomy. Cultures from purulent pleural fluid identified *H. influenzae* and *A. terreus*, necessitating a multidisciplinary approach involving antimicrobials, infectious disease consultation, and pulmonary care. Despite initial improvement, the patient experienced recurrent pyopneumothorax, highlighting challenges in managing complex polymicrobial pleuropulmonary infections. This case underscores the importance of considering rare pathogens in empyema, particularly in patients with chronic pleuropulmonary conditions, and emphasizes the need for close follow-up and tailored therapeutic strategies to optimize outcomes.

## Introduction

Pleural effusions can be classified as transudative or exudative based on pleural fluid analysis. Exudative effusions often result from infections (e.g., pneumonia), malignancies, or autoimmune conditions (e.g., rheumatoid arthritis or lupus). Empyema is a type of exudative effusion characterized by the presence of pus in the pleural space, typically arising from bacterial infections [1]. Rarely, fungal infections or parasitic infections, such as *Entamoeba histolytica*, can cause empyema [2]. Empyema may be classified as parapneumonic (associated with pneumonia) or non-parapneumonic (unrelated to pneumonia) [2]. 

*Streptococcus pneumoniae*, other streptococcal species like *Streptococcus pyogenes*, *Staphylococcus aureus*, oral anaerobes, Enterobacteriaceae, *Pseudomonas aeruginosa*, and mycobacterial species are the most commonly implicated bacteria in parapneumonic effusions [3]. Non-parapneumonic empyema involves a broader range of causative organisms [2]. *Haemophilus influenzae*, a pleomorphic gram-negative rod, is a commensal of the human nasopharynx and a known cause of childhood infections such as empyema, epiglottitis, cellulitis, bacteremia, meningitis, and septic arthritis [4]. While these infections are more common in children, *H. influenzae* can also cause acute COPD exacerbations and non-bacteremic pneumonia in older adults [4]. 

Fungal infections account for less than 1% of pleural effusions, with Candida species the most implicated fungi [2]. Cryptococcus and Aspergillus species are less commonly implicated [2]. Infections caused by Aspergillus species are collectively known as aspergillosis, with common pathogens including *Aspergillus fumigatus, Aspergillu​​​​​​​s flavus, Aspergillus terreus, and Aspergillu​​​​​​​s niger* [5]. These fungi are ubiquitous in soil, air, water, and decaying organic matter [5]. *A. terreus*, an emerging opportunistic fungus, can cause allergic bronchopulmonary aspergillosis, bronchitis, and invasive or disseminated aspergillosis [5]. While invasive pulmonary aspergillosis typically occurs in immunocompromised individuals, pleural aspergillosis and empyema can develop in immunocompetent patients, especially in patients with conditions like tuberculosis, bronchopulmonary fistulas, pleural drainage, and a history of lung resection [6]. 

This report details the case of a patient with chronic obstructive pulmonary disease (COPD) and pleuropulmonary disease caused by untreated rheumatoid arthritis, who developed pyopneumothorax due to dual infections with *A. terreus* and *H. influenzae*. The case highlights the complexity of managing polymicrobial infections and the importance of multidisciplinary care in such conditions.

## Case presentation

The patient was a 70-year-old man with a history of severe emphysematous COPD requiring oxygen therapy, type 2 diabetes mellitus (well controlled on insulin), seropositive rheumatoid arthritis (untreated due to patient refusal), and chronic bilateral hydropneumothoraces. He presented to the emergency department (ED) with a two-day history of increasing oxygen requirements and worsened cough. 

Two days prior to the ED presentation, the patient had seen his pulmonologist, who noted hypoxia with an oxygen saturation of 82%. He had also reported worsening shortness of breath over the preceding weeks and was started on oral doxycycline and prednisone for possible COPD exacerbation and provided with anticipatory guidance. Despite this, his symptoms persisted, prompting his presentation to the ED. His bilateral hydropneumothoraces, present for several years, were attributed to rheumatoid arthritis. A thoracic surgery evaluation a year earlier deemed him unsuitable for surgical intervention for his pleuropulmonary disease. 

**Figure 1:**
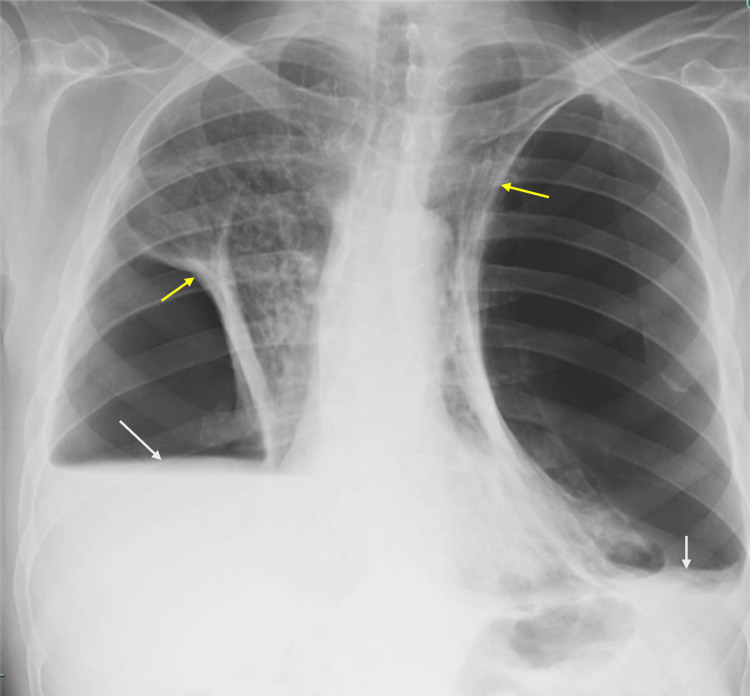
Chest X-ray at admission showed bilateral pleural effusion (white arrows), collapsed lungs (yellow arrows), and pneumothoraces (space between arrows)

In the ED, the patient had oxygen saturations ranging between the 50s and 60s on 3L of low-flow nasal cannula (LFNC), requiring brief escalation to a non-rebreather mask at 15L to stabilize saturations. He was afebrile and hemodynamically stable. Physical exam revealed diminished breath sounds globally and moderate work of breathing on 6L LFNC, with eventual improvement on heated high-flow oxygen. At admission, arterial blood gas showed the following values: pH 7.28, pCO₂ 114 mmHg, pO₂ 82 mmHg, and bicarbonate (HCO₃⁻) 41 mEq/L. Additionally, the patient had a normal white blood cell count. Chest radiography revealed chronic large bilateral hydropneumothoraces, and compared to the examination one year prior, the left side demonstrated improved fluid but worsened gas within the pleural space and lung volume loss, while the right side demonstrated worsened fluid and lung volume loss with improved gas, as seen in Figure 1. The patient's pulmonologist was consulted and recommended continuing doxycycline and prednisone.

**Figure 2:**
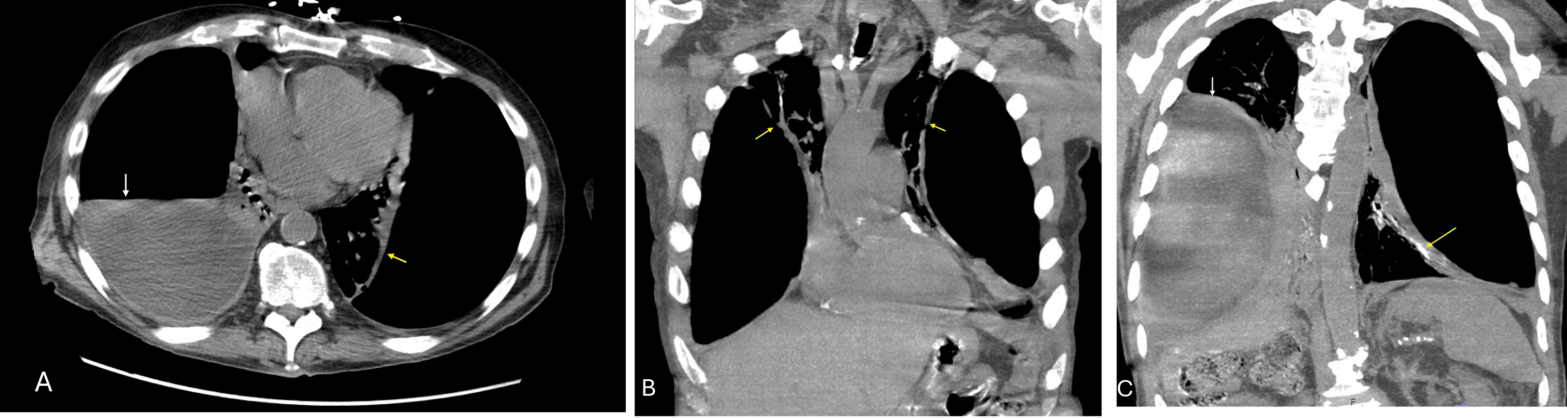
A CT of the chest on day two of hospitalization showed the following: A. Axial reconstruction showing bilateral pneumothorax and collapsed lungs (yellow arrow) with a large right-sided pleural effusion (white arrow). B. Coronal reconstruction showing bilateral pneumothorax with collapsed lungs (yellow arrows). C. Coronal reconstruction showing left pneumothorax with a collapsed lung (yellow arrow) and a right-sided pleural effusion (white arrow) CT: computed tomography

On day two of hospitalization, a computed tomography (CT) scan of the chest revealed large bilateral hydropneumothoraces (Figure 2). The left pleural space was almost entirely gas-filled, while the right showed a significant pleural fluid component, along with marked bilateral lung volume loss and atelectasis. Due to concerns about pneumothorax ex vacuo, chest tube placement was deferred at this time. 

On day three of hospitalization, a 14 French pigtail chest tube was placed percutaneously in the right pleural space due to worsening oxygenation and a white blood cell count increase to 13200/µL. The drained pleural fluid was purulent, with a nucleated cell count of 39440/mm³ and 98% segmented cells. Intravenous cefepime was initiated alongside the patient’s doxycycline. Cultures later identified beta-lactamase-producing strains of *H. influenzae *with fungal growth on day four of hospitalization, prompting fluconazole initiation and cefepime replacement with ceftriaxone following susceptibility results. Infectious disease was consulted, the patient’s immunodeficiency workup was negative, and a four-week antimicrobial plan was established. Despite intermittent air leaks suggesting the presence of bronchopleural fistula, the patient's clinical status gradually improved. 

**Figure 3:**
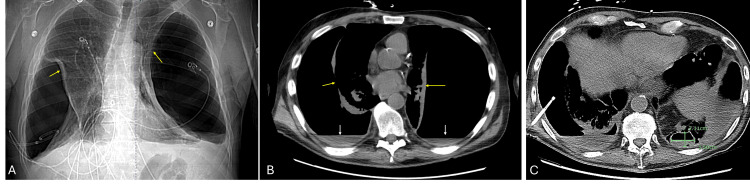
A CT of the chest on day ten of hospitalization following chest tube thoracostomy showed A. A CT scout view showing reduced right pleural effusion, with persistent bilateral pneumothorax and collapsed lungs (yellow arrows); B. An axial reconstruction showing pleural effusion bilaterally (white arrows), with persistent bilateral pneumothorax and collapsed lungs (yellow arrows); C. An axial reconstruction highlighting a 3.5 cm x 2.1 cm cavitary lesion in the left lower lung lobe CT: computed tomography

On day ten of hospitalization, a repeated chest CT scan revealed bilateral hydropneumothoraces with reduced right pleural fluid, increased left-sided pleural fluid, and a 3.5 cm x 2.1 cm cavitary lesion in the left lower lobe (Figure 3). The next day, the patient inadvertently removed his chest tube. He was later discharged on IV ertapenem and oral fluconazole to complete a four-week course. Ertapenem was chosen for its similar activity to that of ceftriaxone against beta-lactamase-producing strains of *H. influenzae*, broader activity against anaerobes, efficacy in polymicrobial infections, and once-daily dosing. Pleural fungal cultures later grew *A. terreus,* leading to fluconazole being switched to itraconazole. 

**Figure 4:**
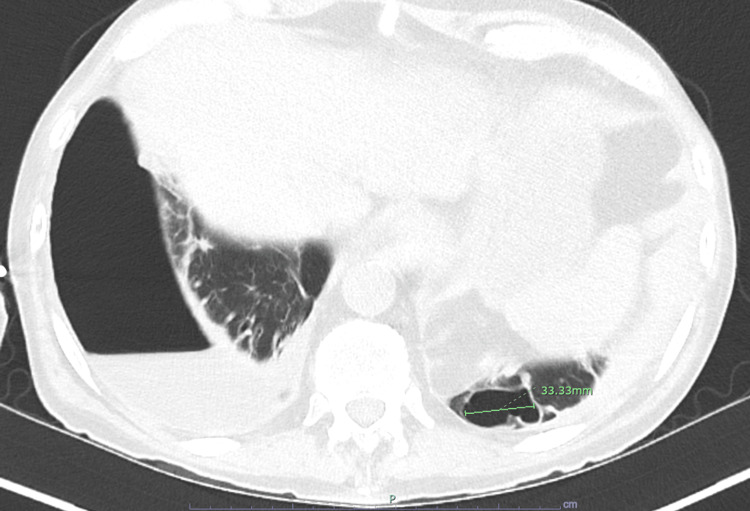
A CT of the chest one month following hospitalization showed resolved internal fluid in the left lower lung lobe cavitary lesion. Right-sided pneumothorax and re-accumulating pleural effusion can also be appreciated CT: computed tomography

A one-month follow-up chest CT scan showed stable findings (Figure 4), with the left lower lobe cavitary lesion still present but with nearly resolved internal fluid. However, the patient was subsequently lost to follow-up, according to his infectious disease provider. 

**Figure 5:**
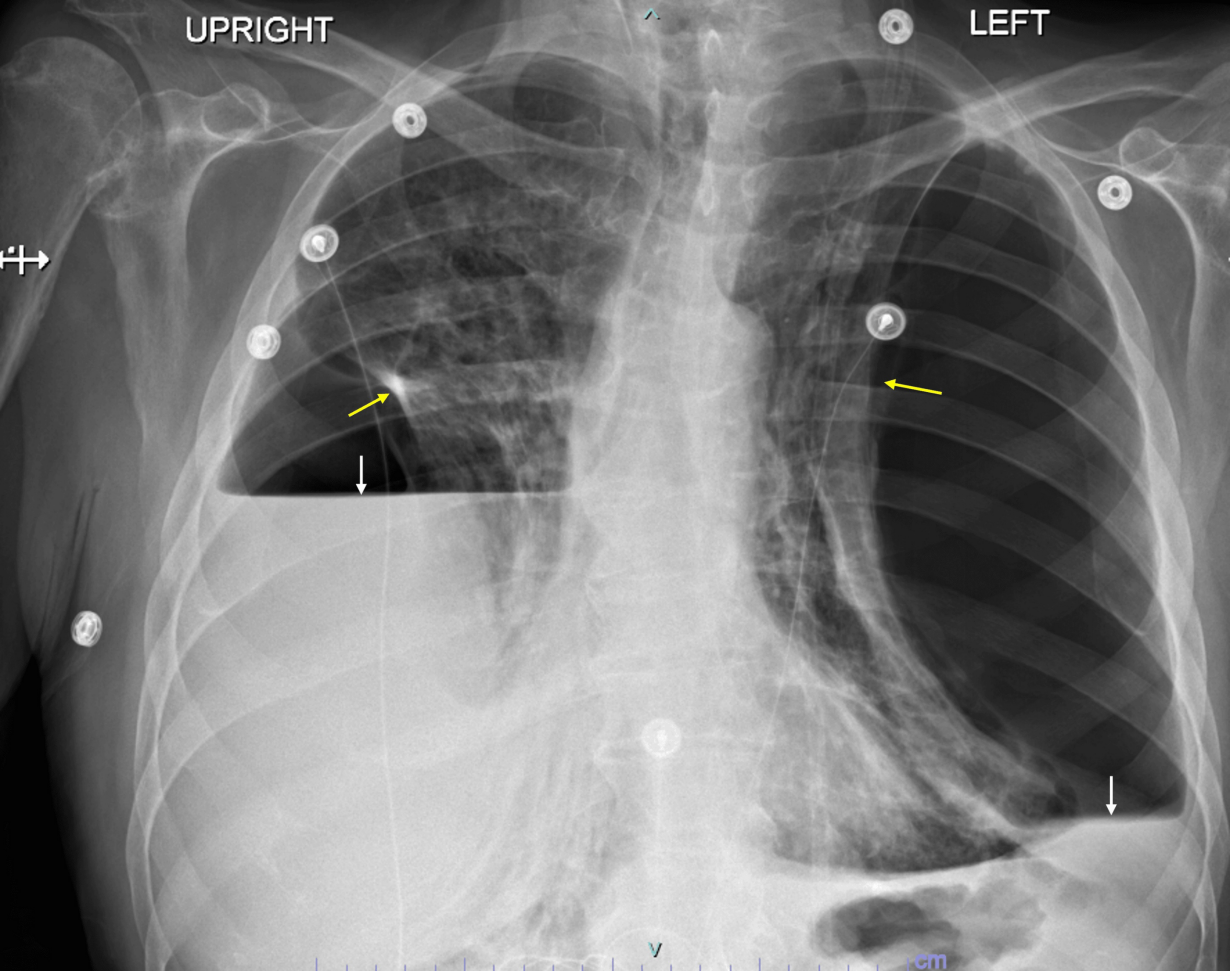
A Chest X-ray showed bilateral pneumothorax and lung collapse (yellow arrows), with bilateral pleural effusion, more pronounced on the right (white arrows)

Two months after his initial admission, the patient was readmitted for sepsis secondary to clostridium difficile infection and treated with oral vancomycin. A month later, he was hospitalized again for worsening dyspnea. Chest X-ray showed reaccumulation of right pleural effusion and persistent bilateral hydropneumothoraces (Figure 5). His white blood cell count was 14000/µL, and he had a low-grade fever. Sputum cultures revealed *Enterobacter cloacae* and *Pseudomonas aeruginosa,* prompting cefepime initiation. Right pleural fluid drainage via a 12 Fr pigtail chest tube identified *Streptococcus anginosus*, *Haemophilus parainfluenzae*, and anaerobes (peptostreptococcus species, capnocytophaga species, and prevotella species). He was later discharged on a course of oral antibiotics. 

## Discussion

*H. influenzae* and *A. terreus* are both uncommon causes of empyema, and their presence in a polymicrobial infection is even rare [4,6]. The patient's comorbidities, such as chronic pleuropulmonary disease due to untreated rheumatoid arthritis and COPD, put him at increased risk for such infections [4,6]. Empyema in patients with chronic hydropneumothorax presents management challenges, as chest tube drainage is often required. However, complications like pneumothorax ex vacuo, due to non-expanding lungs, can arise [7]. Also, if the underlying cause of the chronic hydrothorax is not addressed, pleural fluid often reaccumulates quickly [8]. Another commonly related condition is bronchopleural fistula. These fistulas can both result from empyema and increase the risk of recurrence; additionally, there are known associations between these fistulas and pleuropulmonary infection with *H. influenzae* and Aspergillus* *[9]. These fistulas are more common in individuals over sixty years old or those with chronic conditions like COPD and rheumatoid pleuropulmonary disease [9]. The presence of such fistulas can impair pleural drainage and enable pathogen transfer from the airways, contributing to recurrent infections and complications [9]. During his initial hospitalization, the patient in the case presented was evaluated for interventional or surgical management of his air leaks and possible bronchopleural fistula; however, he was deemed not to be a suitable candidate.

Complex cases of recurrent pyopneumothoraces, like the one discussed, require a multidisciplinary team, including an infectious disease specialist, pulmonologist, thoracic surgeon, and primary care physician. Involving the primary care provider is crucial for improving follow-up and communication between the patient and all healthcare providers [10]. A significant challenge in managing this patient was poor follow-up, as he failed to follow-up with his infectious disease provider after discharge. The patient ultimately received follow-up care during subsequent hospital admissions. 

## Conclusions

This case highlights the importance of considering uncommon pathogens in empyema, even in immunocompetent patients, especially those with chronic pleuropulmonary conditions. Patients with chronic hydrothorax need close monitoring for pleuropulmonary infections due to their increased risk. Additionally, in patients with COPD, the nonspecific nature of COPD exacerbation signs may necessitate evaluation for other pleuropulmonary complications. Finally, treatment strategies should be individualized to ensure patient acceptance and adherence, with an emphasis on proper follow-up.

## References

[1] Shen KR, Bribriesco A, Crabtree T (2017). The American Association for Thoracic Surgery consensus guidelines for the management of empyema. J Thorac Cardiovasc Surg.

[2] Light RW (2007). Pleural Diseases. https://books.google.com/books/about/Pleural_Diseases.html?id=vHEpRHQXaKUC.

[3] Marks DJ, Fisk MD, Koo CY (2012). Thoracic empyema: a 12-year study from a UK tertiary cardiothoracic referral centre. PLoS One.

[4] Khattak ZE, Anjum F (2023). Haemophilus Influenzae Infection. https://www.ncbi.nlm.nih.gov/books/NBK562176/.

[5] Lass-Flörl C (2018). Treatment of infections due to Aspergillus terreus species complex. J Fungi (Basel).

[6] Ko SC, Chen KY, Hsueh PR, Luh KT, Yang PC (2000). Fungal empyema thoracis: an emerging clinical entity. Chest.

[7] Ponrartana S, Laberge JM, Kerlan RK, Wilson MW, Gordon RL (2005). Management of patients with "ex vacuo" pneumothorax after thoracentesis. Acad Radiol.

[8] Balbir-Gurman A, Yigla M, Nahir AM, Braun-Moscovici Y (2006). Rheumatoid pleural effusion. Semin Arthritis Rheum.

[9] Salik I, Vashisht R, Sharma S, Abramowicz AE (2024). Bronchopleural Fistula. https://www.ncbi.nlm.nih.gov/books/NBK534765/.

[10] Rieger EY, Kushner JN, Sriram V, Klein A, Wiklund LO, Meltzer DO, Tang JW (2021). Primary care physician involvement during hospitalisation: a qualitative analysis of perspectives from frequently hospitalised patients. BMJ Open.

